# Severe Hyperbilirubinemia Secondary to Henna Application in a Neonate With G6PD Deficiency: A Case Report and Literature Review

**DOI:** 10.7759/cureus.34540

**Published:** 2023-02-02

**Authors:** Abdulhamid Al-Hinai, Aza S AlSawafi

**Affiliations:** 1 Pediatrics, Oman Medical Specialty Board, Muscat, OMN; 2 Pediatric Medicine, Royal Hospital, Muscat, OMN

**Keywords:** hemolytic anemia, g6pd, henna, neonatal jaundice, hyperbilirubinemia

## Abstract

Henna is a natural product commonly used for cosmetics, healing, and social occasions in the Middle East and South Asian countries. It usually carries no significant medical complications in a healthy individual. However, henna in a patient with G6PD deficiency can cause serious medical complications, including severe hyperbilirubinemia and hemolytic anemia, due to its oxidative stress on the erythrocyte. This paper reports a previously undiagnosed G6PD deficient neonate who presented with severe hyperbilirubinemia without the classical laboratory findings of hemolytic anemia. In addition, we reviewed the literature and summarized the clinical and laboratory findings of 31 G6PD-deficient pediatric patients with henna-induced hemolytic anemia (HIHA). The reported adverse effects of HIHA included death (N: 2), kernicterus (N: 3), life-threatening hemolytic anemia that required blood transfusion (N: 9), and severe hyperbilirubinemia requiring exchange transfusion (N: 7). Although HIHA in G6PD deficiency is a well-known fact in the literature, we believe it is still under-reported. Given the high prevalence of G6PD deficiency and the widespread practice of henna application, we recommend avoiding it, especially in infancy, until the G6PD status is known. Society awareness should be raised about it.

## Introduction

Henna is a natural product obtained from the crushed leaves of Lawsonia alba. Its application to skin and hair for cosmetics, healing, and social occasions is a widely used practice in Oman, like most other Middle East and South Asian countries. The active integrant of henna, lawsone (2-hydroxy-1,4-naphthoquinone), has been linked to henna-induced hemolytic anemia (HIHA). It may, however, be limited to people with weakened antioxidant defenses such as glucose-6-phosphate dehydrogenase (G6PD) deficiency [1].

G6PD enzyme is an essential housekeeping enzyme, especially for erythrocytes, which plays a vital role in preventing cellular damage from reactive oxygen species (ROS). G6PD deficiency is a common X-linked genetic disorder, affecting upwards of 400 million people worldwide. It is more prevalent in people from Asia, Africa, and the Mediterranean, most likely due to its malaria-protective effect [2,3]. In Oman, the prevalence of G6PD is estimated to be 25% in males and 10% in females [4].

In this paper, we report a previously undiagnosed G6PD deficient neonate who presented with severe hyperbilirubinemia without the classical laboratory findings of hemolytic anemia. To define the clinical and laboratory findings of HIHA in G6PD deficiency, we conducted a literature review for all reported pediatric cases of henna application effects in G6PD-deficient patients. A systematic search was implemented using four literature databases (PubMed, Springer Link, Science Direct, and EBSCO Host). The search terms henna, lawsone, and G6PD were used. All reported cases for patients above the age of 13 years were excluded. We found 31 cases reported in 11 papers from different countries, mainly from the Middle East [5-15].

## Case presentation

A one-month-old baby girl presented to our emergency department with two days history of yellowish discoloration of the sclera and face that was noted one day after Henna's application to her palm and sole. The baby, otherwise, remained well. She tolerated breastfeeding and passed a good amount of urine with no change in urine or stool color. The parent declined any history of fever, rashes, upper respiratory tract symptoms, vomiting, and abnormal bowel movements. She had been exclusively breastfed since birth. The family history was unremarkable for any hematological disorder.

The mother of this child was a primigravida who was on regular treatment for immune thrombocytopenic purpura and had regular follow-ups with a hematologist. Her pregnancy was uneventful, with no significant risk factors for sepsis. The baby was delivered at term at 38 weeks of gestation via emergency lower cesarean surgery due to non-progression of labor. Her birth weight was 2.6 kg. She had good APGAR scores of 8 and 9 in 1 minute and 5 minutes, respectively. The neonate's examination was unremarkable. Postnatal lab screening showed a normal platelet count and thyroid-stimulating hormone (TSH) of 5.4 U/mL. The baby was released from the hospital after receiving her birth immunizations (Bacille Calmette-Guerin (BCG) and hepatitis B vaccine).

During this emergency department visit, at age of one month, the baby was found to be icteric. She was hemodynamically stable with good hydration status. She had gained 800 grams since birth. There were no dysmorphic features, cataracts, skin rashes, or hepatosplenomegaly. The neurology examination was unremarkable.

**Figure 1:**
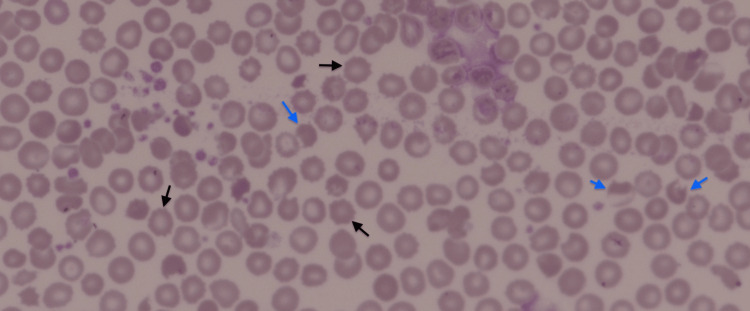
Peripheral blood film from the patient that shows burr cells (indicated by black arrows) as well as bite cells (indicated by blue arrows)

Laboratory examination showed unconjugated hyperbilirubinemia of 397 umol/L (normal range: 5-21). A complete blood count showed a normal hemoglobin concentration of 10.0 g/dL (10-14.1), hematocrit 30.5% (31-55), mean corpuscular volume 91.1 fL (76-96), mean corpuscular hemoglobin concentration 32.7 g/dL (31-35), red cell distribution width 15% (11.5-16.5), platelet count 337* 109/L (150-450), and white blood cells 12* 109/L (6-20). The reticulocyte percentage was only 1.6% and the absolute reticulocyte count was normal at 51.1* 109/L (20-150). Lactate dehydrogenase was within the normal range of 242 iU/L (180-430). At 10.0 mg/L, haptoglobin was low. There were a few burr and crescent cells on the blood film that indicated hemolysis (Figure 1). The direct anti-globulin test was negative. The baby's blood group was B positive and her mother's blood group was O+. The G6PD enzyme assay showed a deficient 184.2 U/L. A liver function test showed no transaminases or evidence of liver dysfunction and albumin of 34 g/L (34-45), alanine transaminase: 14 iU/L (10-49), and alkaline phosphatase 464 iU/L (90-270). 

The baby was admitted to the general ward, during which she was kept under phototherapy (as per the hospital phototherapy guidelines (NICE guidelines) for around 24 hours. After a subsequent repeat test of bilirubin, the level of bilirubin was safely reduced without reducing hemoglobin levels. Following the cessation of phototherapy, there was no rebound of bilirubin, and neurological examinations were normal. The patient was sent home with instructions to avoid the G6PD triggering factors for hemolysis, including henna.

## Discussion

Although henna application or ingestion carries no significant medical complication in a healthy individual, it can cause a serious complication in G6PD deficiency that might go unnoticed [5]. All reported pediatric cases of HIHA in G6PD deficient share the same underlying pathophysiology of oxidative stress-induced hemolytic anemia, with clear laboratory evidence of hemolysis in the form of anemia, reticulocytosis, blood film evidence, and/or high LDH.

The causal relationship of HIHA to G6PD deficiency in our case could be easily missed, given the absent family history, female gender, and non-classical laboratory findings. Hemoglobin, reticulocytes, and LDH were normal in the presence of severe hyperbilirubinemia, which was unexpected. However, the consumption of haptoglobin, raised carboxyhemoglobin > 2%, and blood film evidence of oxidative hemolysis made us clear about the diagnosis.

This observation was similarly made by P. Kumar Nair and S. Al Khusaiby (2003), who reported three neonates with severe hyperbilirubinemia following henna application with normal hemoglobin and reticulocyte. One might be tempted to question whether this is indeed connected to henna, especially since hemolysis might only contribute modestly to hyperbilirubinemia pathogenesis [16].

Table 1 summarizes the clinical and laboratory findings of 31 reported cases (Male 26/31, Female: 5/31) of HIHA in G6PD-deficient children. The age range was (3 days - 11 years). More than 80% of patients presented with jaundice. The median hours of symptoms after the Henna application was 29 hours.

**Table 1:** All reported pediatric cases of HIHA in G6PD deficiency * After applying Henna D: Days, E: Exchange transfusion, F: Female, M: Male, NA: Not available. P: Phototherapy, T: Pack RBC transfusion, Yrs: Years

Study	Year	Number of cases	Gender	Age	Presentation	The onset of symptoms (Hours)*	Hemoglobin (g/dL)	Bilirubin (mg/dL)	Reticulocyte %	LDH (iU/L)	Haptoglobin (mg/L)	Treatment	Complication
Kandil et al. [5]	1996	15	M	NA	Jaundice/anemia	NA	11.2	382.8	13.4	NA	NA	NA	NA
Raupp et al. [6]	2001	1	F	20 D	jaundice	24	6.9	700	NA	NA	NA	ET	Nil
		2	M	60 D	jaundice, shock	48	2.8	231	NA	2322	NA	T	Died
		3	M	3 yrs.	Pallor, vomiting	72	7.1	132	NA	827	NA	T	Nil
		4	F	4 yrs.	Pallor, vomiting	48	4	168	NA	NA	NA	T	Nil
Kumar Nair, Al Khusaiby [8]	2003	1	M	15 D	Jaundice/sepsis	NA	NA	521	NA	NA	NA	ET	Died
		2	M	3 D	Jaundice	NA	NA	377	NA	NA	NA	ET	Kernicterus
		3	M	5 D	Jaundice	NA	NA	524	NA	NA	NA	ET	Kernicterus
Murat Söker [7]	2001	1	M	11 yrs.	Pallor	NA	4.2	36.2	6.2	721	NA	T	Nil
A.N. KOK et al. [9]	2004	1	M	11 yrs.	Pallor and vomiting	6	6.7	136	10	NA	NA	T	Nil
		2	F	7 yrs.	Pallor and vomiting	6	5.7	205	12	NA	NA	T	Nil
Katar et al. [10]	2007	1	M	7 D	jaundice/ pallor	29	12	994	7	NA	NA	P/ET	Nil
Abolhassan S, Mitra H [11]	2007	1	M	42 D	Jaundice/ pallor	NA	6	136	9.8	NA	NA	T	Nil
Ilkhanipur and Hakimian [12]	2013	1	M	35 D	jaundice/pallor	72	4.7	856	5.5	NA	NA	ET	Kernicterus
Kheir et al. [13]	2017	1	M	6 yrs.	Jaundice/anemia	48	4	51	5.1	NA	NA	T	Nil
Alhazmi A et al [14]	2018	1	F	120 D	Jaundice/fever	24	6.4	222	5.5	NA	NA	T	Nil
Khefacha et al. [15]	2021	1	M	4 D	Jaundice/poor feeding	NA	12	454	454	1548	NA	P+ ET	Nil
Our case	2022	1	F	30 D	Jaundice	24	10	397	1.6	242	<10	P	Nil

The median bilirubin level was 304 mg/dL. Fifty-three percent (53%) of patients were treated with packed red blood cell (PRBC) transfusion, 41% required exchange transfusion, and 1.7% were treated with phototherapy.

The complication of HIHA in the pediatric group was age-dependent. During the neonatal period, it mainly causes hyperbilirubinemia without significant anemia and outside the neonatal period, it presents with severe hemolytic anemia. The outcome of reported cases included death secondary to sepsis (N: 2), kernicterus (N: 3), life-threatening hemolytic anemia (N: 9), and severe hyperbilirubinemia requiring exchange transfusion (N:7).

The effect of henna on G6PD deficiency might be as fast as hours or late as days. With the application practice of henna, one would expect a rebound effect following phototherapy or exchange transfusion; however, this is not the case. This might be due to the higher activity of G6PD in fresh reticulocytes [17] or the bioavailability of the henna gradient in serum following its application, which needs further research.

## Conclusions

The literature recognizes HIHA in G6PD deficiency, but we believe it continues to be underreported. Considering the high prevalence of G6PD deficiency and the widespread practice of henna application, the authors emphasize the need to avoid henna application, especially during infancy, until the screening test is conducted. To avoid unanticipated serious consequences, the report recommends that henna avoidance be made a regular medical recommendation.
